# Description of a double centrifugation tube method for concentrating canine platelets

**DOI:** 10.1186/1746-6148-9-146

**Published:** 2013-07-22

**Authors:** Anna Perazzi, Roberto Busetto, Tiziana Martinello, Michele Drigo, Daniela Pasotto, Francesco Cian, Marco Patruno, Ilaria Iacopetti

**Affiliations:** 1 Department of Animal Medicine, Production and Health, University of Padova, Padova, Italy; 2 Head of Department of Animal Medicine, Production and Health, University of Padova, Padova, Italy; 3 Department of Comparative Biomedicine and Food Science, University of Padova, Padova, Italy; 4 Department of Veterinary Medicine, University of Cambridge, Cambridge, UK

**Keywords:** Dog, Double centrifugation, Growth factors, Platelet-rich plasma, Tube method

## Abstract

**Background:**

To evaluate the efficiency of platelet-rich plasma preparations by means of a double centrifugation tube method to obtain platelet-rich canine plasma at a concentration at least 4 times higher than the baseline value and a concentration of white blood cells not exceeding twice the reference range. A complete blood count was carried out for each sample and each concentrate. Whole blood samples were collected from 12 clinically healthy dogs (consenting blood donors). Blood was processed by a double centrifugation tube method to obtain platelet concentrates, which were then analyzed by a flow cytometry haematology system for haemogram. Platelet concentration and white blood cell count were determined in all samples.

**Results:**

Platelet concentration at least 4 times higher than the baseline value and a white blood cell count not exceeding twice the reference range were obtained respectively in 10 cases out of 12 (83.3%) and 11 cases out of 12 (91.6%).

**Conclusions:**

This double centrifugation tube method is a relatively simple and inexpensive method for obtaining platelet-rich canine plasma, potentially available for therapeutic use to improve the healing process.

## Background

Growth factors are the conductors or modulators of wound healing process through induction of chemotaxis, stimulation of mitosis and upregulation of protein production [1-3]. They enhance tissue healing by stimulating cell proliferation, increasing extracellular matrix synthesis, and promoting vascular ingrowth [4]. Platelet concentrates are an important source of autologous GFs [5]. The platelets α-granules provide a concentrated source of PDGF, TGFβ and numerous other growth factors such as VEGF and PDAF, thromboxane and fibronectin/vitronectin [6]. Platelet rich plasma (PRP) has emerged as an economical method to provide a source and delivery mode for autologous growth factors and cell-rich fractions. It has been suggested that the supraphysiological GFs concentrations present in this substance could act positively by accelerating wound healing, decreasing the inflammatory reaction and promoting the regeneration rather than repair of affected tissues [7,8]. Platelet-rich plasma represents an emerging biotechnology in current tissue engineering and cellular therapy [9]. It has applications in management of extensive skin wounds, and in orthopaedic diseases, primarily for tendon and ligament repair, and cartilage resurfacing [6]; its biocompatible and biodegradable properties prevent the PRP from inducing foreign body reactions, tissue necrosis or extensive fibrosis [10]. Platelet concentrates can be obtained by means of at least three general methods: the apheresis, buffy coat, and tube methods [11]. The apheresis method is performed in completely closed disposable system: an apheresis unit was used to obtain platelet concentrate by a process of discontinuous centrifugation [2]. Setup and processing times for the apheresis method were longer than for the buffy coat method. The retail unit and disposables costs were greater for the apheresis method [2]. Several manufacturers provide specific disposable devices, packaged with syringes, needles and anticoagulant to obtain PRP using the buffy coat method. The separation of the buffy coat and plasma uses differential centrifugation and a specific density floating shelf to separate the bulk of the red blood cell mass from the small white blood cells, platelets and plasma. The platelet-enriched plasma is concentrated by centrifugation immediately above the buffy coat layer of white blood cells [6]. The possibility of bacterial contamination of concentrates may be greater when using buffy coat methods rather than apheresis methods [2]. The tube method has been used for preparing platelet rich plasma; the advantages of this technique are the low cost and the minimal technical requirements compared to the other two general methods described above. However extreme care should be taken to avoid bacterial contamination when this technique is performed [11]. Increasing consent is accumulating that PRP products must achieve a platelet count of at least 300 × 10^3^ platelets μl^-1^ to be in the therapeutically effective range [8,12], indicating approximately a 4–5 times the baseline value [8,9,13-15]. The collective literature would suggest that platelet concentration of 4 times is at or near maximal stimulation, and that leukocyte concentrations should be kept to a minimum to maximize matrix synthesis and minimize inflammation after PRP injection for the treatment of injuries [4]. High WBC counts are unacceptable in blood preparations used for hemotherapy as the result of increased transfusion reactions, they are generally accepted in platelet concentrates used for autologous topical application or as an addictive to a bone graft. It is a matter of speculation as to whether they are beneficial or deleterious to wound healing [2]. The aim of this report is to evaluate the efficiency of PRP preparation using a double centrifugation tube method, relatively simple and inexpensive, to obtain a platelet rich plasma with a platelet concentration of at least 4 times the baseline value and a concentration of white blood cells that do not exceed 2 times the reference range.

## Methods

### Dogs

After obtaining owner consent, twelve clinically healthy dogs were included in this study. All animals were taken at the Department of Department of Animal Medicine, Productions and Health (Faculty of Padua).

### Blood collection

Sixteen millilitres of whole blood were taken from each dog from the cephalic vein using a 21 gauge butterfly needle and divided into two parts, both collected into sodium citrate tubes (BD Vacutainer system, Plymoth, UK).^a^ For each dog a hemogram was performed. The average time between blood extraction and the start of sample processing was 20 min.

### Double centrifugation tube method

Sixteen millilitres of citrated blood were centrifuged (Labofuge 400, Heraeus Holding, Hanau, Germany)^b^ without applying a brake for 20 min at 2800 rpm to achieve separation of cell layers. This procedure divides the blood into three basic components: red blood cells, platelet rich plasma (PRP) and platelet poor plasma (PPP). Red blood cells were isolated from the overlying buffy coat and plasma by the gel-like plug within the tubes. From each tube (8 ml of blood) thus yielded approximately 4–5 mL of platelet-poor plasma, of which 80% was discarded. The buffy coat of each tube, containing mononuclear cells and platelets, was then carefully removed with a pipette and resuspended in 0.75-1.0 mL of the plasma remaining. The final solution, obtained by mixing different buffy coats in a sterile 15 ml Falcon tube, was centrifuged without applying a brake at 1300 rpm for 15 min for good separation of platelet pellets in the supernatant layer. The platelet pellet accumulates at the bottom of the tube, the PPP on top. The PPP is drawn off so that the PRP remains in the tube. After resuspending the platelet pellet within the remaining volume of plasma with the vortex mixer, the final PRP can be drawn up with a syringe. Each step of the method was carried out using sterile disposables.

### Platelet and leukocyte counts

A complete hemogram was performed for each sample using a flow cytometry hematology system (ADVIA 120 Analyzer, Bayer Lab, NY, USA).^c^ Both samples of whole blood and PRP were examined at the end of the procedure. Hematological parameters analyzed included: packed cell volume (PCV), platelet count (PLT), leukocyte count (WBC), as well as the relative and absolute numbers of neutrophils, lymphocytes, monocytes, eosinophils, basophils and large unstained cells (LUC) counts, values for MPV, large platelet count and platelet clumps (aggregates). For all samples, a manual blood film examination was performed in order to confirm the results and evaluate the cell morphology.

### Bacteriological tests

This study was performed using sterile disposables although laminar flow hood has not been used. The choice not to use a laminar flow hood is related to the desire to evaluate the real risk of contamination to verify the reproducibility of this method even in structures that are not equipped with such equipment. For each samples bacteriological tests were carried out in order to verify the level of contamination on sample of PRP obtained at the end of the processing.

### Statistical analysis

All the data obtained were analysed by a commercial statistical software (SPSS for Windows-Release 12.0.1; SPSS Inc., IL USA)^d^. For each measurement of the whole blood, simulated data were calculated according to the target of the procedure (i.e. at least 4 concentration times for platelet count and no more than 2 concentration times for WBC count). Normality of data was assessed by one sample Kolmogorov-Smirnov non parametric test. The Paired samples *t*-test was used to compare whole blood, final PRP and simulated PRP groups. The level of significance was set for p<0.05.

## Results and discussion

Twelve clinically healthy half-breed dogs were included in this study, 6 male and 6 female, aged from 1.5 to 7 years (mean age 3.73 ± 1.74 years). Data for each parameter considered of whole blood, platelet rich plasma and platelet rich plasma simulated are described and summarized in the Table 1; since data are normally distributed, they are expressed as mean ± standard deviation.

**Table 1:** Descriptive results of study variables (data presented as means±standard deviations)

**Variables**	**Whole blood (mean +/- SD)**	**Platelet-rich plasma (mean +/- SD)**	**Simulated platelet-rich plasma (mean +/- SD)**
Red blood cells (cells × 10^6^/μl)	5.72 ± 0.62	0.11 ± 0.06	NA
Lymphocytes (cells × 10^3^/μl)	3.72 ± 0.81	12.87 ± 5.26	NA
White blood cells (cells × 10^3^/μl)	12.35 ± 2.45^a^	14.82 ± 6.57^a^	24.69 ± 4.89^b^
Platelets (cells × 10^3^/μl)	394 ± 18^a^	1981 ± 114^b^	1575 ± 718.5^c^

NA = Not available. ^a,b,c^Letters: significant differences between groups (one-sided p<0.05).

**Figure 1:**
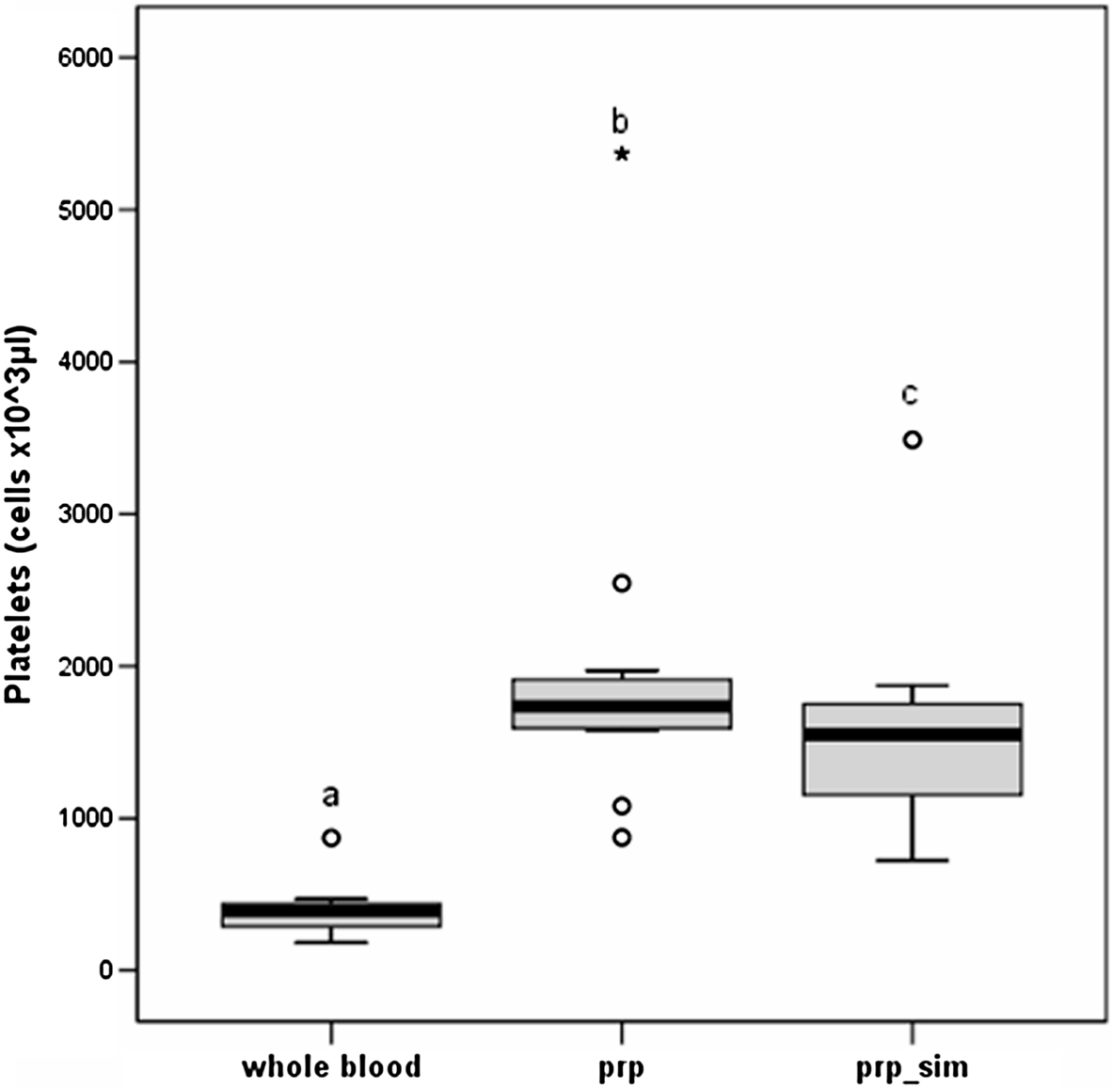
**Boxplots for platelet cytometric count of whole blood, PRP and simulated PRP groups. **^a,b,c^Letters: significant differences between groups (one-sided p<0.05). See Table 1 for descriptive data.

**Figure 2:**
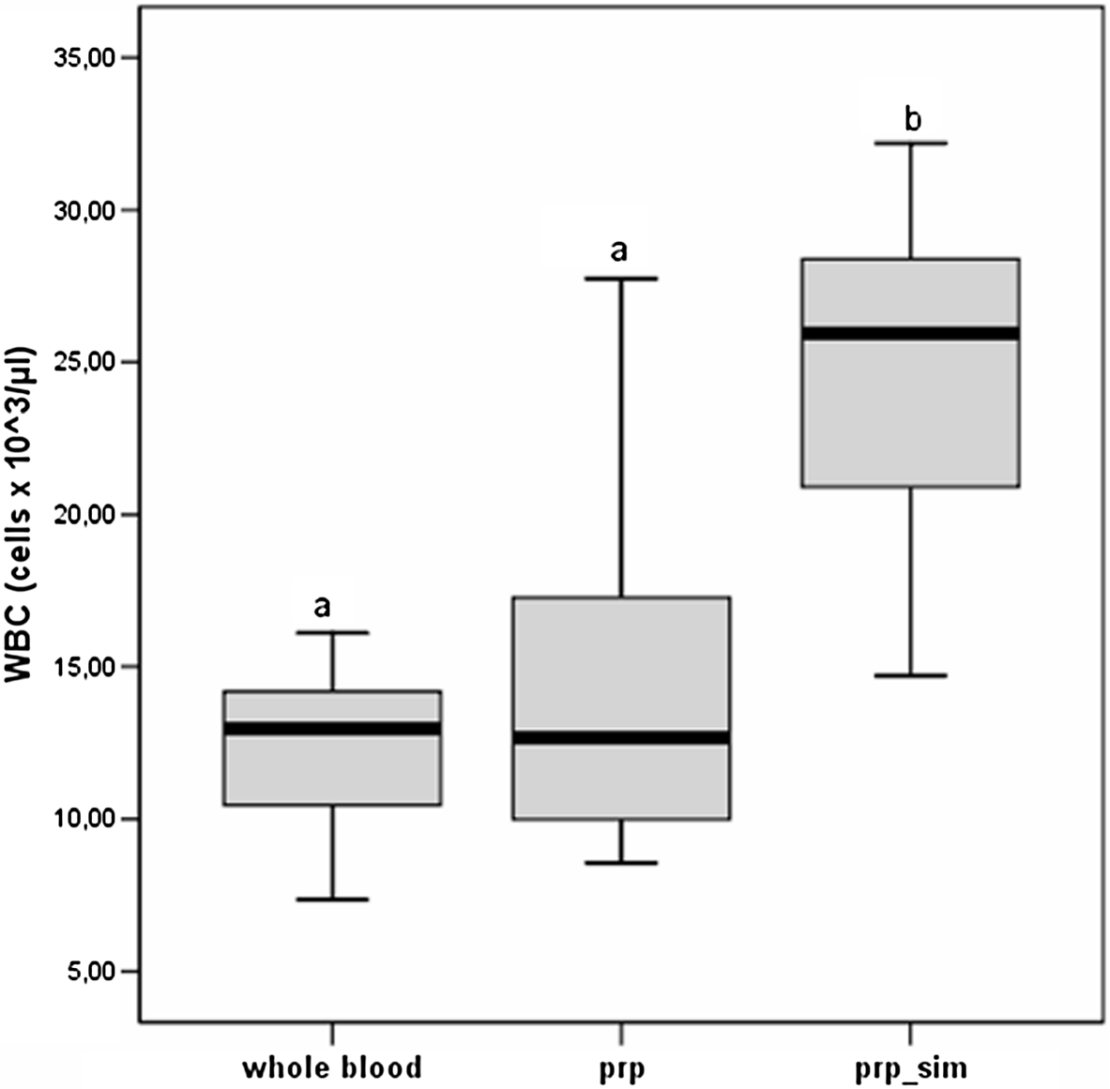
**Boxplots for WBC cytometric count of whole blood, PRP and simulated PRP groups. **^a,b^Letters: significant differences between groups (one-sided p<0.05). See Table 1 for descriptive data.

It is important to note that in the platelet rich plasma the concentration of red blood cells is almost negligible and that 88 ± 5% of white blood cells is represented by lymphocytes. In platelet rich plasma was obtained a mean value of platelet’s concentration of 1981 ± 114 cells × 10^3^/μl. One of the aims set by the authors was to obtain a platelet’s concentration at least 4 times the baseline value. This target was achieved in 10 out of 12 dogs (83.3% of the sample); in the other 2 samples the platelet’s concentration is respectively 3.69 and 3.91 times the baseline value. The cytometric count of platelet is significantly different, from the whole blood, in platelet rich plasma but not in the platelet rich plasma simulated, as evidenced by boxplots below (Figure 1). Furthermore, in platelet rich plasma was obtained a mean value of WBC’s concentration of 14.82 ± 6.57 cells × 10^3^/μl. The other aim set by the authors was to obtain a WBC’s concentration no more than 2 concentration times the baseline value. This target was achieved in 11 out of 12 dogs (91.6% of the sample); in the other sample the WBC’s concentration is 2.08 times the baseline value. The cytometric count of WBC in platelet rich plasma was not significantly different from whole blood; they both differ from the platelet rich plasma simulated, as evidenced by boxplots below (Figure 2).

The first tube method protocol for preparing human PCs intended for alveolar-maxillary reconstruction was originally described by Anitua [11]. There are many papers trying to characterize and classify the numerous techniques available on the market in terms of preparation (centrifugation speed and use of anticoagulant), content (platelets, leucocytes, and growth factors), and applications. Currently, all these aspects are strongly discussed in the literature, and there is no consensus [16]. There is no evidence of standardization of PRP preparation and use [16] but common clinically available materials for blood preparations combined with a two-step low force centrifugations, to ensure cellular component integrity, provided good platelet preparations [17]. In human medicine, the centrifugation steps should be as delicate as possible but allowing a clean separation of the blood components. Performing two successive low force centrifugations can contribute to platelet preservation [17]. Other author described a protocol based on two successive centrifugations to obtain highly concentrated platelets from whole blood: a first spin, also called soft spin, separates plasma, buffy coat (containing platelets), and red blood cells and a second spin or hard spin, further concentrates platelets [16]. The human tube centrifugation protocols are different from the canine tube technique described here since the effect of sedimentation on canine platelets is different from the same effect on human platelets [18]. High centrifugation forces can lead to activation of platelets during the preparation and thus impair or diminish platelet function and activity on the wound directly [17]. However, as well described from Clemmons, when the size difference between platelets and RBCs is greater, separation on PRP at high gravitational force resulted in a good platelet yield both with short or long centrifugation time [18].

Our method involves a double centrifugation: a first hard spin separates plasma, buffy coat (containing PRP), and red blood cells and a second soft spin optimizes platelet concentration without damaging the cells; this method provides the time of centrifugation of the blood that will produce an appropriate enrichment of platelets without being damaged in their morphology.

The high centrifugation force can presents some technical inconveniences that are related to the platelet activation during platelet concentrates preparation. The platelet activation in fact can contribute to the development of platelet storage lesions and decrease platelet viability. It is therefore important to develop a method to minimize platelet activation during the preparation of platelet concentrates [19]. As described by Segawa in 2012 the addition of prostaglandin E_1_ (PGE1) in PRP before the centrifugation of PRP can improve the preparation efficiency of platelet concentrates from dogs, while maintaining the therapeutic efficacy of the platelets [19]. It would be interesting to evaluate the method here described in another group of dogs studying whether the addition of PGE improve the yield and effectiveness of platelets reducing the percentage of activation. Marx [8] postulated that the ideal platelet concentrate preparation method should be simple and easy to perform, with the platelets easily separated from the red blood cells and sequestered in high concentrations without suffering damage so that they can actively secrete their growth factors. The method here described is proved to be quick (about 90 minutes) and simple to be performed and does not require particularly expensive equipment. It is also an easily applicable method in clinical routine. The technique does not require highly qualified staff to run it in an optimal way. As reported by other authors, more simplified PRP preparation methods which do not require ad hoc and costly equipment would help in accumulating clinical data and in introducing the method more routinely in clinical practice as also in the operating room [17]. The tube method presents some technical inconveniences that are related to the risk of bacterial contamination during the preparation of platelet concentrate. It is recommended to use a laminar flow chamber and to have trained sanitary personnel to prepare the platelet concentrate especially when canine platelet concentrates will be used therapeutically [20]. However this would made the technique accessible only by laboratories with this equipment. In our study it has not been possible to work commonly using a laminar flow chamber, but during all phases of production the personnel have worked, dressed in lab coats, headgear, mask and sterile gloves, using sterile disposables on washable surfaces appropriately disinfected. For each sample bacteriological tests were carried out in order to verify the level of contamination on sample of PRP obtained at the end of the processing. Samples of PRP resulted sterile on bacteriological tests in 11 cases out of 12 (91.67%). In one sample were found 3 colonies of *coagulase-positive Staphylococcus*; this level of contamination should be considered minimum. This result is satisfactory and makes this method ideal for using the platelet concentrate for therapeutic purposes in regions that do not necessarily require a close asepsis, such as the oral cavity, skin and eye. Given the excellent results we obtained in terms of sterility of the samples, to use this technique for therapeutic purposes in regions that require a close asepsis it may be sufficient to perform first a bacterial culture on the platelet rich plasma sample obtained to use the product with a greater safety margin. Increasing consent is accumulating that PRP products must achieve a platelet count of at least 300 × 10^3^ platelets μl^-1^ to be in the therapeutically effective range [8,12], indicating approximately a 4–5 times the baseline value [8,9,13-15]. In our study a platelet concentration of at least 4 times the baseline was obtained in 10 out of 12 dogs (83.3% of cases). In the remaining 2 samples the platelet’s concentration is respectively 3.69 and 3.91 times the baseline value: these values are still very close to the target set by the study. Moreover in absolute terms, the PRPs of these 2 subjects contained a concentration of platelets greater than 300 × 10^3^ platelets μl^-1^ (respectively 1725 × 10^3^ and 1763 × 10^3^ platelets μl^-1^). The collective literature would suggest that leukocyte concentrations should be kept to a minimum to maximize matrix synthesis and minimize inflammation after PRP injection for the treatment of injuries [4]. In the samples of platelet rich plasma, white blood cells count lower than 2 times the reference range was obtained in 11 out of 12 dogs (91.6% of cases). The real role of the leukocytes present in human or animal platelet concentrates prepared for tissue healing has never been clarified very well and their true effect is completely unknown [16]. It should be considered, for example, that when equine platelet concentrate was injected intra-articularly in horses with severe joint diseases [20], joint effusion and inflammation were observed during the first days of post treatment. These clinical effects could be explained by the chemotactic effect of the leucocytes present in high concentrations in that platelet concentrate. There is a still speculation on the effect of leukocytes [7] in the PRP used to stimulate healing. Leukocytes greater than those of the blood are generally acceptable in autologous platelet concentrates [21,22]. In human medicine the transfusion of platelet concentrates are used primarily in homologous transfusion therapy of patients with severe thrombocytopenia and/or platelet’s disorders. It is known that such transfusion may cause side effects caused by leukocytes that remain in such preparations such as immunization-HLA, refractoriness to platelet transfusion reactions, febrile non-haemolytic transfusion or other complications [21,23]. For the prevention of such complications, in human medicine it has been proposed for several years, to transfuse platelets with a number of WBC < 5 × 10^5^[24]. There are no data to ensure whether the action of white blood cells is beneficial or not to tissue repair [6,25]. A high number of leucocytes could produce an inflammatory response in certain tissues, such as articular tissue. Fortier recommends choosing a PRP with a high concentration of platelets and a low concentration of leukocytes, so as to maximize anti-inflammatory effects of TGF-β and reduce catabolism [4]. The influence of leukocytes on the biology of each product and its potential benefits in term of healing tissue should be analyzed in more detail in order to explain the many controversial data of literature [25].

## Conclusion

In conclusion, the double centrifugation tube method may represent a relatively simple and inexpensive method for obtaining canine platelet rich plasma potentially available for therapeutic use. Our method has to be evaluated on a higher number of samples, but the positive preliminary results achieved suggest this technique might effective and easily applicable. The procedure can be standardized and is easy to adapt in clinical settings with minimal infrastructure, thus enabling large numbers of subjects to benefit from a form of cellular therapy.

## Availability of supporting data

The data sets supporting the results of this article are included within the article.

## Endnotes

^a^BD Vacutainer system, Plymouth, UK.

^b^Labofuge 400, Heraeus Holding, Hanau, Germany.

^c^ADVIA 120 Analyzer, Bayer Lab, NY, USA.

^d^SPSS Inc., IL, USA.

## Abbreviations

PRP: Platelet-rich plasma; PRG: Platelet-rich gel; GFs: Growth factors; PDGF: Platelet-derived growth factor; TGFβ: Transforming growth factor; VEGF: Vascular endothelial growth factor; PDAF: Platelet-derived angiogenesis factor; WBC: White blood cells; PPP: Platelet-poor plasma; PCV: Packed cell volume; LUC: Large unstained cell; MPV: Mean platelet volume; PCs: Platelet concentrates; HLA: Human leukocyte antigen; RBC: Red blood cells.

## Competing interests

The authors declare that they have no competing interests.

## Authors’ contributions

AP and II conceived of the study and participated in its design and coordination and helped to draft the manuscript. TM has made substantial contributions to conception and design. DP carried out the bacteriological tests. FC participated in the analysis and interpretation of data. MD performed the statistical analysis. MP have been involved in drafting the manuscript. RB proofread the manuscript and has given final approval of the version to be published. All authors read and approved the final manuscript.

## Authors’ information

This manuscript is part of a PhD thesis submitted by Dr. Perazzi to the School of Veterinary Medicine, University of Padova, Italy.
